# Cross recurrence quantification analysis of precision grip following peripheral median nerve block

**DOI:** 10.1186/1743-0003-10-28

**Published:** 2013-03-02

**Authors:** Ke Li, Zong-Ming Li

**Affiliations:** 1 Hand Research Laboratory, Departments of Biomedical Engineering, Orthopaedic Surgery, and Physical Medicine and Rehabilitation, Cleveland Clinic, Cleveland, Ohio, USA

**Keywords:** Median nerve block, Precision grip, Hand, Cross recurrence quantification analysis, Phase synchronization, Nonlinear dynamics

## Abstract

**Background:**

Precision grip by the thumb and index finger is vulnerable to sensorimotor deficits. Traditional biomechanical parameters offer limited insight into the dynamical coordination between digits during precision grip. In this study, the thumb and index finger were viewed as “coupled systems”, and a cross recurrence quantification analysis (CRQA) was used to examine the changes of interdigit dynamics and synchronization caused by peripheral median nerve block.

**Methods:**

Seven subjects performed a precision grip by holding an instrumented handle before and after median nerve block at the wrist. The forces and the torques at each digit-handle interface were recorded with two six-component transducers. For CRQA, the percentage of recurrence rate (%RR), percentage of determinism (%DET), longest diagonal line (Lmax) and percentage of laminarity (%LAM) were computed for the force, torque and center of pressure (COP) signals. Phase synchronization of the thumb and index finger was examined based on the *τ*-recurrence rate. Paired *t*-tests and Wilcoxon signed-rank tests were used for statistical comparisons. The twin-surrogate hypothesis test was used to examine phase synchronization.

**Results:**

Nerve block led to significant increases (*p* < 0.05) for %DET, Lmax and %LAM in all components of force, torque, and COP. Only the normal force met the conditions of phase synchronization for all successfully completed pre- and post-block grasping trials. The probability of synchronization with larger time lags (*τ* > 0.1 s) increased after nerve block. The percentage of trials that the thumb led the index finger increased from 52% (pre-block) to 86% (post-block).

**Conclusions:**

Nerve block caused more deterministic structures in force, torque and COP when the thumb interacted with the index finger. A compensatory mechanism may be responsible for this change. Phase synchronization between the opposite normal forces exerted by the thumb and index finger would be an essential dynamical principle for a precision grip. The nerve block resulted in an increased interdigit phase delay and increased probability that the thumb leads the index finger. The CRQA provides an effective tool to examine interdigit coordination during precision grip and has the potential for clinical evaluation of hand dysfunction.

## Background

Precision grip plays an important role in a variety of daily activities, such as lifting, holding, and handwriting. The seemingly effortless precision grip requires sophisticated spatial and temporal coordination of the digit forces that can adapt to object properties and movement states. This delicate coordination is vulnerable to a number of central or peripheral neurological lesions, such as Parkinsonism [1], stroke [2] and impaired tactile sensibility [3].

Nerve block provides an effective way to transiently interrupt the sensorimotor system and simulate hand dysfunctions caused by peripheral neuropathies. It was observed that peripheral nerve block impaired grip and pinch strength [4] and thumb abduction and flexion strength [5]. Fine motor control (e.g. precision grip) can also be disrupted by nerve block. The deterioration of precision grip in individuals with peripheral median nerve block involves inaccurate “pulp-to-pulp” contact [6], excessive grip force [7] and a larger migration area of each digit’s center of pressure (COP) [8]. These findings improve our understanding of some irregular mechanics of precision grip, yet more research is needed to examine how the thumb dynamically coordinates or discoordinates with the index finger during object manipulation.

The anatomical and neural arrangement of the thumb and index finger substantiates the two digits as interdependent systems that strongly couple and intelligently match each other during grasping [9]. Their three-dimensional (3-D) forces and torques are under control of both feedforward and feedback mechanisms. The feedforward mechanism allows individuals to program the appropriate motor commands prior to grasping according to previous experiences of the object properties; whereas the feedback mechanism adjusts gripping according to real-time sensory information [9]. However, little knowledge exists about how these mechanisms are involved in the dynamical control of an individual digit or in the digit positioning for object manipulation. It is of interest to study the interaction of the two digit systems during precision grip in order to better understand the dynamical neural control mechanisms for fine object manipulation.

The time series of the forces and the torques from the thumb and index systems by nature are highly complex, exhibiting nonlinear and nonstationary characteristics. Such complexity is due to the integration of both the feedforward and the feedback motor control mechanisms which regulates the manual manipulation of objects. Exploration of the inherent information of these complex motor systems requires suitable analytical tools. Traditional time- or frequency-analyses (e.g. Fourier transform or coherence analysis) have limitations regarding the analyses of nonstationary, low-frequency kinetic signals [10,11]. Some dynamical measures (e.g. fractal dimensions or Lyapunov exponents) were developed for univariate signals with long-range variability, but are limited in quantifying nonlinear interrelations from short bivariate and nonstationary time series [12]. Recently, the cross recurrence quantification analysis (CRQA) has been introduced as an advanced technique for nonlinear, neurophysiological signals. CRQA provides a group of statistical parameters to analyze the structures of a cross recurrence plot (CRP). The CRP is a graphical representation of a matrix whose elements correspond to all the moments when phase-space trajectories of one system pass through the neighborhoods of trajectories of another system [13]. CRQA is particularly suitable for the study of interdigit coordination as it is capable of revealing the interactions of two dynamical systems with robustness against model presumption, nonstationarity transients, outliers, and noise that often limit the use of other methods [14]. In addition, CRQA is an effective tool to analyze the phase synchronization (PS) of two coupled dynamical systems [15]. PS means that phases or frequencies of two chaotic systems become locked, even though their amplitudes remain uncorrelated [13]. PS has been observed in human cognition and behaviors, such as neuron activities [16], corticomuscular coupling [17], or binocular eye movements [13]. However, it has yet to be determined whether the thumb and index finger synchronize in their kinetic signals during a precision grip.

The aim of this study was to examine the dynamical coordination of the thumb and index finger during precision grip using CRQA, and to identify the inherent changes in the underlying kinetic signals caused by peripheral median nerve block. It was hypothesized that the dynamical structures of the forces, torques, and/or COPs, and the mutual relationships of the PS between the thumb and the index finger would be altered by nerve block.

## Methods

### Experiment data

The experimental data used for the current analyses was adopted from our previous study [8]. Seven healthy male subjects (26.9 ± 5.1 years old) participated in the experiment. A peripheral median nerve block was achieved by injecting 4 mL of 0.5% bupivacaine hydrochloride (Astra Pharmaceuticals, Westborough, MA, USA) into the carpal tunnel. Nerve block was confirmed by the Semmes-Weinstein monofilament test with a pre-block score of 2.85 (calculated force: 0.0677 g) across the five digits and post-block scores of 6.15 (127.0 g) in the median nerve distribution and 3.22 (0.1660 g) in the ulnar nerve distribution. The apparatus used in the experiment included two miniature six-component force/torque transducers (Nano17, ATI Industrial Automation, Inc., Apex, NC, USA) that were mounted on a custom-made rectangular aluminum handle (size: 15 × 80 × 130 mm; weight: 341 g). The grip-contact surfaces of the transducers had a diameter of 17 mm and were covered with 180 grit sandpaper. The distance between the two gripping surfaces was 44 mm.

During the experimental trials, subjects were asked to use the pads of their thumb and index finger to grip the surfaces of the transducers, then lift and hold the handle as stably as possible for 60 s. Subjects were instructed to hold the handle vertically to minimize tilting. In order to eliminate the effects of visual feedback, subjects turned their head to the left to avoid looking at the handle during holding. Before the formal testing, each subject was given several practice trials to be familiarized with the handle properties and the test protocol. Although unsuccessful grasping trials randomly occurred for some subjects, each subject was instructed to follow a consistent test protocol without discernible changes in experimental setup, feedback condition or grasping posture between the successful and aborted trials. Each subject successfully completed the task three times before and three times after nerve block. Three forces (F_x_, F_y_, and F_z_ in the x-, y-, and z-axes) and three torques (T_x_, T_y_, and T_z_ around the x-, y-, and z-axes) were recorded from transducers for the thumb and index finger with a sampling frequency of 500 Hz. The coordinate system of each transducer was aligned with respect to a common coordinate system by multiplying by a rotation matrix. The x- and y-axes were the vertical and horizontal directions in the transducer’s surface plane, respectively, and the z-axis was perpendicular to the surface plane.

### Data analysis

#### Preprocessing

The instantaneous COP at each digit-object interface was calculated using the torques around the x- and y-axes of the grip surfaces (T_x_ and T_y_) and the normal force (F_z_). The coordinates of the COP (P_x_, P_y_), with respect to each transducer’s coordinate frame, were calculated as P_x_ = −T_y_/F_z_ and P_y_ = T_x_/F_z_. The three force components (F_x_, F_y_, F_z_), three torques around the x-, y- and z-axes (T_x_, T_y_, T_z_), and two COP coordinates (P_x_, P_y_) from the thumb transducer were each paired with its counterpart from the index transducer. These signals were low-pass filtered with a cutoff frequency of 20 Hz. This frequency band is considered to be the physiologically meaningful spectrum for kinetic signals during a sustained isometric contraction [11,18]. For each signal pair, the holding phase from 10–60 s was retained for CRQA.

#### Cross recurrence quantification analysis

CRQA was applied to quantify changes in the regularity and spatiotemporal properties for each of the eight (F_x_, F_y_, F_z_, T_x_, T_y_, T_z_, P_x_, P_y_) signal pairs between the thumb and index finger for all successful grasping trials. Using CRQA, the following four parameters were derived: the percentage of recurrence rate (%RR), the percentage of determinism (%DET), the longest diagonal line (Lmax), and the percentage of laminarity (%LAM) [13]. The %RR quantifies regularity by calculating the probability of occurrence of similar states in two systems [13]. Greater %RR corresponds to greater correlation in a time series [19]. The %DET is the percentage of recurrence points that form diagonal structures to all recurrence points in the CRP. If both systems have similar phase space behavior, the number of longer diagonals increases and the number of shorter diagonals decreases, resulting a higher%DET [13]. Therefore, %DET reflects the deterministic or predictable structure between two dynamical systems [20]. The Lmax represents the longest diagonal line found in the CRP. It is related to the exponential divergence of the phase space trajectory and correlation entropy [13]. The %LAM quantifies the density of recurrent points that form vertical line structures in the recurrence map. It demarcates time intervals during which the system’s state is relatively constant compared to intervals of sudden bursts of activity [13,20]. CRQA was performed using an embedding dimension of 1 [21], a time delay of 1 sample [22], and a threshold setting to 10% of the maximum phase space radius [13]. Parameters of CRQA were implemented with the cross recurrence plot toolbox 5.16 of MATLAB (The Mathworks, Natick, MA, USA).

#### Phase synchronization analysis

The synchronization of two dynamical systems can be visualized by a line of synchronization (LOS) plotted on the CRP [13]. The LOS segments that are parallel to the main diagonal reveal the time series synchronization at a time instant; while those deviating from the main diagonal show the phases or frequencies of the two systems that were unlocked at that time [23]. Therefore, LOS provides visualization of phase shifts or frequency variations between two systems [13,23].

A synchronization index (SI) is defined based on CRQA [13]:

(1) $$\mathrm{SI}=〈p^{\vec{x}}\left(\tau \right)p^{\vec{y}}\left(\tau \right)〉$$

where the *p*(*τ*) is the recurrence rate for the diagonal lines within distance *τ* of the main diagonal on the CRP.

$$p^{\vec{x}}\left(\tau \right)$$

and

$$p^{\vec{y}}\left(\tau \right)$$

represent the maxima of *p*(*τ*) for two time series x and y, and the < > denotes the computation of correlation coefficient. The SI value ranges from 0 to 1. *p*(*τ*) is estimated as the *τ*-recurrence rate (*RR*_*τ*_):

(2) $$p\left(\tau \right)=RR_{\tau }=\frac{1}{N-\tau }\sum_{i=1}^{N-\tau }\Theta \left(\varepsilon -\left\|\vec{x_{i}}-\vec{x_{i+\tau }}\right\|\right)$$

If both systems are in PS, *p*(*τ*) of the two signals achieves maxima simultaneously and thus the SI approaches 1. By contrast, if systems are not in PS, the maximum *p*(*τ*) of each signal does not occur simultaneously, leading to a SI value far less than 1.

However, use of SI alone, even if has a value close to 1, is insufficient to ascertain the systems are in PS. A twin-surrogate hypothesis test is an essential inspection to avoid any potential interferences of series randomness or system noise on the SI results. A surrogate should be an independent realization of one of the original systems, such as a mimic system maintaining the same trajectories or attractors as the original one, but with different initial conditions. By comparing the SI calculated from the original system with those calculated from surrogates, a hypothesis test is able to validate the status of PS statistically [13]. In our study, the hypothesis test worked on 100 surrogates for all time series of the force (F_x_, F_y_, F_z_), torque (T_x_, T_y_, T_z_), and COP (P_x_ and P_y_). Only if the SI of the original signal pair is significantly higher (95% confidence interval [CI], *p* < 0.05) than those of the surrogates can the null hypothesis be rejected, and the two signals be accepted as in PS for that trial. The probability of PS was calculated as the percentage of trials that met the conditions of PS.

For the signals that reliably synchronized from trial to trial, tests based on CRQA were further performed: (a) to calculate the average time delay of one digit system with respect to the other, and (b) to determine which of the systems (i.e. thumb and index finger) more frequently leads the other. The tests were based on the measure *RR*_*τ*_ in (2), which is often calculated as

(3) $$RR_{\tau }=\frac{1}{N-\tau }\sum_{l=1}^{N-\tau }lP_{\tau }\left(l\right)$$

where *l* is the length of the diagonal lines and *P*_*τ*_(*l*) is the number of diagonal lines within a distance *τ* (0 ≤ *τ* ≤ N) above the main diagonal line on the CRP. The recurrence-rate within the same distance *τ*, but below the main diagonal line, can be represented as *RR*_−*τ*_. Then the measures of symmetry and asymmetry are defined, respectively, as (4) and (5):

(4) $$Q_{1}\left(\tau \right)=\frac{RR_{\tau }+RR_{-\tau }}{2}$$

(5) $$Q_{2}\left(\tau \right)=\frac{RR_{\tau }-RR_{-\tau }}{2}$$

When *Q*_1_(*τ*) reaches its maximum, the input value represented as *τ*, or an integer multiple of *τ*, indicates the average time lag between the two synchronized systems. At the moment when *Q*_1_(*τ*) reaches maxima, the sign of *Q*_2_(*τ*), either positive or negative, indicates which of the two signal inputs leads the other. For example, if *Q*_2_(*τ*) > 0, the first signal leads the second signal with a lag *τ*, and vice versa [13]. The *τ* value was preset at 0.05 s in (4) and (5).

#### Statistical analyses

Statistical analyses were performed using SPSS (SPSS Inc., Chicago, IL). The mean and 95% CI of each CRQA parameter were calculated for the conditions before and after nerve block. The normality of CRQA measures was verified by skewness and kurtosis, as well as the Kolmogovrov-Smirnov test. For a measure with a normal distribution, a paired *t*-test was used to examine the effects of nerve block. A Wilcoxon signed-rank test was used to evaluate the effects of nerve block for a measure that was not normally distributed. PS was examined for each signal pair using the SI and the twin-surrogate hypothesis test (95% CI). All the trials that fulfilled PS were pooled together to examine the distribution of the time lags and the probabilities of one digit leading the other one. A *p*-value of less than 0.05 was considered statistically significant.

## Results

**Figure 1:**
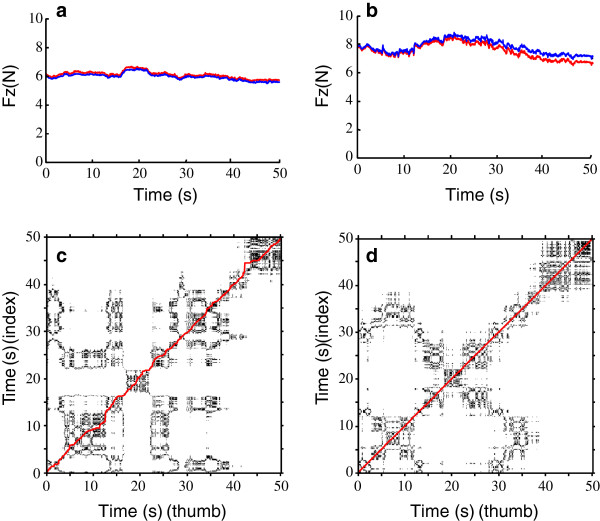
**Normal forces and their CRPs for a representative subject before and after nerve block.** (**a**) F_z_ of the thumb (red line) and index finger (blue line) before nerve block; (**b**) F_z_ of the thumb and index finger after nerve block; (**c**) CRP of (**a**); (**d**) CRP of (**b**); The red diagonal line on (**c**) and (**d**) are lines of synchronization (LOS).

Normal forces (F_z_) and the corresponding CRPs for a representative participant are depicted in Figure 1. The post-block force showed a higher magnitude in comparison with the pre-block force. Both pre- and post-block forces presented slight tendencies of nonstationary fluctuations and interdigit inequity (Figure 1a and b). The CRPs of both pre- and post-block data (Figure 1c and d) showed some mutual characteristics: (1) “nonuniformity”, particularly a fading pattern towards the upper left and lower right corners from 40 s to 50 s, reflecting increased divergence from each other; (2) “disruptions”, meaning that some states irregularly recur with transitions; and (3) “cyclicities”, particularly along and close to the main diagonal line, revealing quasi-periodic processes. Unlike the pre-block CRP, the post-block CRP presented some structures parallel to the main diagonal line around 30–40 s (Figure 1d). This indicates the evolution of states between two signals during that period of time (30-40 s) similar to that at a previous time (0-10 s).

**Figure 2:**
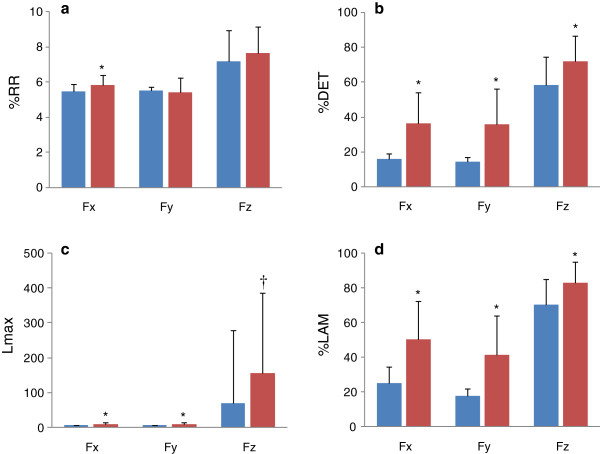
**CRQA parameters for 3**-**D forces before and after nerve block.** (**a**) %RR; (**b**) %DET; (**c**) Lmax; (**d**) %LAM. Data are means ± 95% CI. Blue: pre-block measures; red: post-block measures. * indicates significant difference using paired *t*-test; † indicates significant difference using Wilcoxon signed-rank test.

**Figure 3:**
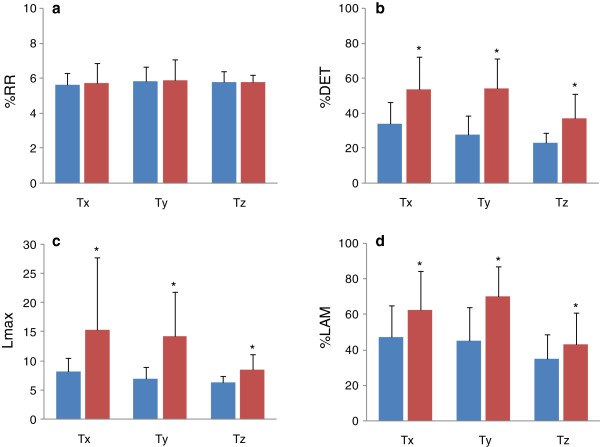
**CRQA parameters for 3**-**D torques before and after nerve block.** (**a**) %RR; (**b**) %DET; (**c**) Lmax; (**d**) %LAM. Data are means ± 95% CI. Blue: pre-block measures; red: post-block measures. * indicates significant difference.

**Figure 4:**
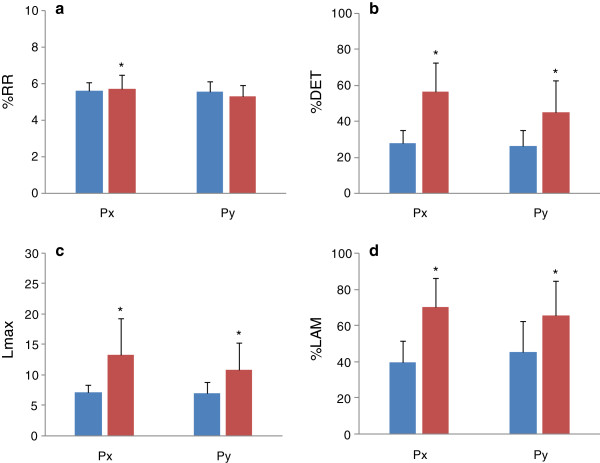
**CRQA parameters for COPs before and after nerve block.** (**a**) %RR; (**b**) %DET; (**c**) Lmax; (**d**) %LAM. Data are means ± 95% CI. Blue: pre-block measures; red: post-block measures. * indicates significant difference.

Statistical analyses showed that all of the examined parameters, except for Lmax of F_z_, were normally distributed. Results of CRQA for forces, torques, and COPs are presented in Figures 2, 3, and 4. In comparison to the pre-block results, post-block data did not show significant changes in %RR for F_y_ (*p* = 0.541) and F_z_ (*p* = 0.078), but did for F_x_ (*p* < 0.05) (Figure 2a). For %DET, Lmax, and %LAM, significantly higher values were found in all three force signals after nerve block (*p* ≤ 0.001, Figures 2b,c, and d). The %DET, Lmax, and %LAM were also found to be significantly increased in T_x_, T_y_, and T_z_ after nerve block (*p* < 0.01, Figures 3b, c, and d). %RR did not differ significantly in T_x_ (*p* = 0.776), T_y_ (*p* = 0.881), or T_z_ (*p* = 0.992) (Figure 3a). Nerve block caused significant increases in %DET, Lamx, and %LAM for P_x_ and P_y_ (*p* < 0.01, Figures 4b, c, and d), but not for P_y_ (*p* = 0.138, Figure 4a) in %RR.

The synchronization of the thumb and index finger systems during sustained precision grip was examined both qualitatively and quantitatively. The LOSs of the normal forces (F_z_) were extracted from the corresponding CRPs (Figure 1c and d). The LOS approximately fit to the main diagonal of the CRP for both the pre- and post-block data, but some distortions were discernible representing thumb-index finger asynchrony at those moments (Figure 1c). Table 1 presents the SI scores and probabilities of PS according to the twin-surrogate hypothesis test. Among all signals, only F_z_ fully met the conditions of PS (100% trials, with high SI values close to 1) before and after nerve block (Table 1). This revealed that the normal forces of thumb and index finger reliably synchronized regardless of whether or not the nerve was blocked.

**Table 1:** **SI and probability of PS for all interdigit signal pairs for the pre**- **and post**-**block conditions**

**Signal pair**		**F**_**x**_	**F**_**y**_	**F**_**z**_	**P**_**x**_	**P**_**y**_	**T**_**x**_	**T**_**y**_	**T**_**z**_
**Mean of SI**^**(a)**^	**Pre**	0.49	0.47	**0**.**95**	0.72	0.68	0.78	0.68	0.69
	**Post**	0.83	0.77	**0**.**97**	0.84	0.78	0.80	0.87	0.72
**SD of SI**^**(b)**^	**Pre**	0.23	0.23	**0**.**03**	0.13	0.16	0.12	0.17	0.17
	**Post**	0.20	0.27	**0**.**02**	0.16	0.19	0.21	0.17	0.28
**Probability**^**(c)**^	**Pre**	76%	76%	**100%**	81%	81%	86%	81%	90%
	**Post**	90%	86%	**100%**	81%	86%	71%	76%	81%

(a) Mean value of the SI for all trials; (b) Standard deviation of the SI for all trials; (c) Percentage of trials that fulfilled the criteria of PS by the twin-surrogate hypothesis test; Bolded and underlined values show that only the Fz had high SI values and completely fulfilled the criteria of PS for all trials.

**Figure 5:**
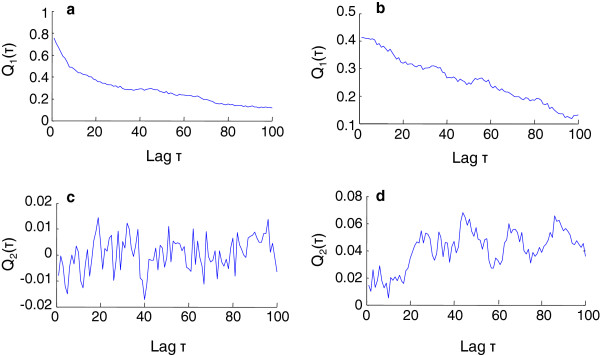
**Examples of *Q***_**1**_(*τ*) **and *Q***_**2**_(*τ*) **calculated from the CRPs in Figure**1**.** (**a**) and (**b**) *Q*_1_(*τ*) calculated from the CRPs of Figure 1c and Figure 1d, respectively; (**c**) and (**d**) *Q*_2_(*τ*) calculated from the CRPs in Figure 1c and Figure 1d, respectively. The maxima of *Q*_1_(*τ*) were observed at *τ* = 1 for the both trials ((**a**) and (**b**)), revealing signals of the thumb and index finger of these trials were synchronized with a time lag of less than 0.05 s. *Q*_2_(*τ*) is negative in (**c**), but positive in (**d**) at *τ* = 1, suggests that the first system led the second system in (**c**), but lagged behind the second system in (**d**).

Figure 5 presents the *Q*_1_(*τ*) and *Q*_2_(*τ*) that were calculated from the F_z_ of the two grasping trials shown in Figure 1a and b, with a time lag *τ* changing from 1 to 100 times. The maxima of *Q*_1_(*τ*) was observed at *τ* = 1 for the both trials, indicating that the thumb and finger systems synchronized with a time delay of no more than 0.05 s. At the moment *τ* = 1, a negative *Q*_2_(*τ*), discernable in Figure 5c, revealed that the index finger led the thumb during holding. However, the positive *Q*_2_(*τ*) in Figure 5d at *τ* = 1, showed a contrasting relationship that the thumb led the index finger for this trial.

**Figure 6:**
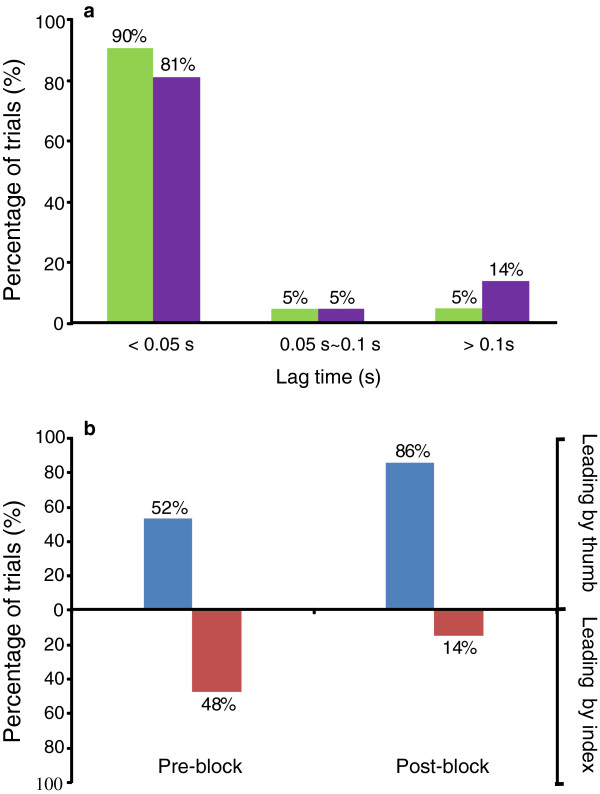
**Phase synchronization between digits**: **phase delay and leading or lagging relationships.** (**a**) Percentage of trials when the thumb and index finger synchronized with different lags before (green) and after nerve block (purple). (**b**) Percentage of trials that the thumb led the index finger (blue) and the index finger led the thumb (red) before and after nerve block.

The probabilities of PS according to F_z_ before and after nerve block are shown in Figure 6a. For the pre-block condition, 90% of the trials showed that the thumb synchronized with the index finger at a time delay within 0.05 s; 5% of the trials had synchronization with a lag between 0.05 s and 0.1 s; and the remaining 5% of the trials had a lag greater than 0.1 s (Figure 6a). After the nerve block, the probability of PS with a time lag of less than 0.05 s decreased to 81%, but the probability of a time lag of greater than 0.1 s increased to 14% (Figure 6a). Figure 6b further illustrates the probabilities of the leading or the lagging relationships between the two digits. The thumb led the index finger system in 52% of the trials. After nerve block, the thumb preceded the index finger in 86% of the trials, and only in 14% of the trials did the index finger precede the thumb (Figure 6b).

## Discussion

Precision grip is a common daily activity that requires intricate coordination between the digits. Traditional assessments of precision grip using the grip or load force typically calculated from all of the involved digits are unable to reflect the activity of individual digits, and are insufficient to comprehensively understand the interdigit dynamical coordination. By means of CRQA, this study examined the interdigt dynamical coordination during precision grip before and after nerve block from two aspects: (1) the dynamical structure of the digit systems and (2) the interdigit phase synchronization. Before and after nerve block, the thumb and index finger systems performed at a similar recurrence rate (%RR), but with different diagonal (%DET and Lmax) and vertical (%LAM) patterns in CRPs (Figures 2, 3 and 4). The increased %DET indicates a more deterministic structure of interdigit interaction after nerve block. The increased Lmax after nerve block is a sign of decreased correlation entropy and increased attractor strength. This suggests that after nerve block the interdigit coordination had less of a chaotic behavior, but stronger attractor dynamics [13]. The higher%LAM represents the increased occurrence of laminar states in both systems, meaning more vertical structures than single points were exhibited in the post-block CRP [13]. This result indicates reduced probability of unstable periods in finger coordination after nerve block for the non-dropping trials. It is worth noting that similar changes were also found in forces, COPs and torques (Figures 2, 3, and 4). These results suggest that nerve block changed the dynamical coordination of the thumb and index finger during a precision grip by systematically raising deterministic structures in all prehensile kinetic signals.

A compensatory mechanism underlying the control strategy of grip may be responsible for the dynamical changes caused by nerve block. Control of precision grip involves both feedforward mechanisms that exert grip force in anticipation of external loads, and feedback mechanisms that regulate grip force based on signals from the mechanoreceptors [24]. Under the nerve block condition, sensory feedback was intensely obstructed, increasing the potential of instability or even failure during grasping. Correspondingly, the task of maintaining a stable hold evoked a compensatory mechanism to reinforce the feedforward motor control. A higher magnitude of grip force and an enlarged safety margin have been observed after nerve block and could be attributed to this compensatory mechanism [8]. In this study, increased CRQA measures after nerve block suggest that strengthened feedforward motor commands under compensatory mechanisms render a more deterministic interdigit coordination. Interestingly, similar dynamical changes have been recognized in other motor behaviors, showing the reinforced deterministic structures of chaotic systems as a sign of functional degradation; such examples include postural instability in Parkinson’s disease [25] and cardiovascular autonomic dysfunction in diabetes mellitus [26]. By contrast, decreased %DET and Lmax were reported in the power grip strength of patients with metabolic disorders [27], indicating deterministic structures could be weakened in some cases. A possible explanation for this inconsistency is that the low force level in precision grip leaves a large amount of motor units available under the action of compensatory mechanism; yet, the exhausted motor units’ recruitment during power grip prevents further compensatory adjustment [28]. The compensatory mechanism thereby is task-specific, depending on whether it is under maximal voluntary contraction (e.g. power grip) or submaximal effort (e.g. precision grip).

Stable grip requires that the digits apply optimal and coordinated forces to form an equilibrium state. In this study, the signal pair of F_z_ from the thumb and index finger met the conditions of PS across all successful grasping trials, even under the nerve block condition (Table 1). This finding suggests that, despite weakened tactile afferents or motor control caused by nerve block, the force components perpendicular to the two digit-object contact surfaces reliably synchronized with each other in the form of PS throughout the holding period. This PS was achieved even though there were mismatched amplitudes of forces, torques and COPs between the individual digits. Therefore, it seems that the PS between the opposite normal forces exerted by the involved digits is an essential dynamical principle of a precision grip.

This PS-principle, however, would be partially influenced by the nerve block. Firstly, the time lag of synchronization tends to prolong after nerve block. The probability of synchronization with a time lag *τ* of less than 0.05 s decreased from 90% before nerve block to 81% after nerve block, whereas that with a lag *τ* of greater than 0.1 s increased from 5% to 14% after nerve block (Figure 6a). Increased time lag raises the risk of asynchrony between the thumb and index finger, which may ultimately lead to a grasping failure. The increased asynchrony helps explain the experimental observation that some subjects dropped the handle at least once after nerve block, despite applying a higher compensatory grip force [8]. This observation suggests that decreased tactile sensitivity or improper motor commands caused by nerve block may destroy the precision grip by disrupting the phase synchrony between digits.

The second influence of the nerve block pertains to the leading or lagging relationship between the digits (Figure 6c and d). Before nerve block, the thumb and index finger had approximately equal probabilities (52% vs. 48%) of taking the leading position. After nerve block, the thumb more frequently led the index finger (86% vs. 14%). This finding suggests that under compensatory mechanism, the thumb plays a more dominant role than the index finger in maintaining interdigit synchronization. Reasons associated with this change may include the anatomical (e.g. finger configuration or muscle volume) or functional (e.g. range of motion) differences between the thumb and the index finger [29].

In this study, the CRQA and peripheral median nerve block were jointly applied to investigate the precision grip. CRQA provided an effective way to disclose the abundant inherent information about the interdigt dynamical coordination underlying prehensile kinetics, such as deterministic or stochastic components, structural complexity, periodic patterns, or phase synchronization [13]. This dynamical information is to a large extent independent of force magnitudes that may be easily interfered by handle orientation. Therefore, CRQA is an analytical tool to indentify functionally meaningful actions in fuzzy, complex, and dynamic behaviors [13]. The median nerve block enabled realization of a transient perturbation in healthy subjects through the interruption of the hand sensorimotor system, which mimics symptoms of inflammatory or noninflammatory polyneuropathies or carpal tunnel syndrome [5,6]. Methods applied in this study will help us understand the mechanisms of digit interaction, and facilitate the diagnosis of sensorimotor deficits in individuals with peripheral neuropathies.

## Conclusions

Nerve block changes the dynamical coordination of the thumb-index finger systems for a precision grip. More deterministic structures were found in kinetic signals after nerve block. A compensatory feedforward mechanism may be responsible for this change. Static object holding requires PS between the two digits, which could be reliably observed from the normal forces. Nerve block led to an increase in average time delay between two synchronized digits and an augment of probability that the thumb leads the index finger. CRQA is a technique that enables qualitative and quantitative examination of the dynamical changes in coupled systems. Further studies are needed to examine the correlation between CRQA parameters and the severity of hand disorders in different populations. This may facilitate the development of a method for clinical diagnosis of multiple peripheral neuropathies that impair hand sensorimotor function.

## Abbreviations

CI: Confidence interval;COP: Center of pressure;CRP: Cross recurrence plot;CRQA: Cross recurrence quantification analysis;Fx Fy, Fz: Force in the x-, y-, and z-axes;Tx Ty, Tz: Torques around the x-, y-, and z-axes;Px Py: Coordinates of center of pressure;Lmax: Longest diagonal line;LOS: Line of synchronization;PS: Phase synchronization;%DET: Percentage of determinism;%LAM: Percentage of laminarity;%RR: Percentage of recurrence rate;Q1(τ): Symmetry measure at specific time delay *τ*;Q2(τ): Asymmetry measure at specific time delay *τ*;RRτ: *τ*-recurrence rate;SI: Synchronization index

## Competing interests

The authors declare that they have no competing interests.

## Authors’ contributions

KL designed computer programs, analyzed and interpreted data and wrote the manuscript. ZML designed the study, collected data, and co-wrote the manuscript. All authors read and approved the final manuscript.

## Authors’ information

KL is a postdoctoral research fellow in the Department of Biomedical Engineering, Lerner Research Institute, Cleveland Clinic, OH, USA. KL is also a faculty member in the Department of Biomedical Engineering, School of Control Science and Engineering, Shandong University, China. ZML is an associate professor at Cleveland Clinic Lerner College of Medicine and an associate staff at the Departments of Biomedical Engineering, Orthopaedic Surgery, and Physical Medicine and Rehabilitation at the Cleveland Clinic, Cleveland, OH, USA.
